# The guanine exchange factor SWAP70 mediates vGPCR-induced endothelial plasticity

**DOI:** 10.1186/s12964-015-0090-1

**Published:** 2015-02-15

**Authors:** Julie Dwyer, Sandy Azzi, Héloïse M Leclair, Steven Georges, Agnès Carlotti, Lucas Treps, Eva M Galan-Moya, Catherine Alexia, Nicolas Dupin, Nicolas Bidère, Julie Gavard

**Affiliations:** CNRS, UMR8104, 22 rue Mechain, 75014 Paris, France; INSERM, U1016, 22 rue Mechain, 75014 Paris, France; Universite Paris Descartes, Sorbonne Paris Cite, 6 rue de l’Ecole de Medecine, 75006 Paris, France; Service de Pathologie, Hopital Cochin-Tarnier, AP-HP, Paris, France; Inserm UMR_753, Institut Gustave Roussy, Villejuif, 94800 France; Service de Dermatologie, Hopital Cochin-Tarnier, Assistance Publique-Hôpitaux de Paris AP-HP, Paris, France

**Keywords:** Angiogenesis, Human herpes virus 8, Kaposi sarcoma herpes virus, Permeability, Rac, Small GTPases

## Abstract

**Background:**

The viral G protein-coupled receptor (vGPCR) is proposed to act as one of the predominant mediators of Kaposi’s sarcoma (KS), a human herpes virus 8 (HHV8)-elicited disease. The actions of vGPCR manifest pathogenesis, in part, through increased permeability of endothelial cells. Endothelial cell-cell junctions have indeed emerged as an instrumental target involved in the vasculature defects observed within the tumor microenvironment. The pathway leading to adherens junction destabilization has been shown to involve the activation of the small GTPase Rac, in the context of either latent infection or the sole expression of vGPCR. However, the precise molecular mechanisms governed by vGPCR in vascular leakage require further elucidation.

**Findings:**

Guanine exchange factors (GEFs) function as critical molecular switches that control the activation of small GTPases. We therefore screened the effects of 80 siRNAs targeting GEFs on vGPCR-driven endothelial permeability and identified switch-associated protein 70 (SWAP70) as necessary for its elevating effects. Pull-down experiments further showed that Rac activation by vGPCR was dependent on SWAP70. Examination of tissues and cells from HHV8-positive patients revealed that SWAP70 was ubiquitously expressed. Furthermore, SWAP70 was found to be crucial for vGPCR-driven endothelial tube formation and endothelial sprouting *in vitro*.

**Conclusions:**

SWAP70 appears to act as a molecular intermediate between vGPCR and endothelial activation. Because of the important role of vGPCR-mediated endothelial plasticity in KS pathogenesis, inhibition of SWAP70 function could be of interest for blocking vGPCR-driven activities in HHV8-defined diseases.

**Electronic supplementary material:**

The online version of this article (doi:10.1186/s12964-015-0090-1) contains supplementary material, which is available to authorized users.

## Findings

Of the multiple genes encoded by the human herpes virus 8 (HHV8), the viral G protein-coupled receptor (vGPCR) is one of the key instigators of Kaposi’s sarcoma (KS) pathogenesis [1]. vGPCR indeed exhibits pleiotropic actions in the manifestation of diseases such as KS and primary effusion lymphoma (PEL), among which are paracrine transformation and endothelial defects [2-7]. This constitutively active homologue of the IL-8 chemokine receptor CXCR2 initiates and propagates a KS-like phenotype in mice engineered to express the gene in an endothelial-restricted manner [2,8,9]. From a molecular standpoint, we have previously demonstrated that vGPCR exploits the IL-8 pro-permeability pathway, which involves the activation of the small GTPase Rac [4,10,11]. In line with this, Rac signaling has been reported to act upstream of endothelial permeability in latently infected-endothelial cells [6]. Moreover, Rac also controls vGPCR-mediated chemokine and pro-angiogenic secretion [12], therefore placing Rac at the cornerstone of vGPCR molecular piracy. Consequently, interruption of vGPCR/Rac actions could hamper disease progression in HHV8-infected individuals. However, how vGPCR orchestrates Rac activation in endothelial cells is unknown.

**Figure 1:**
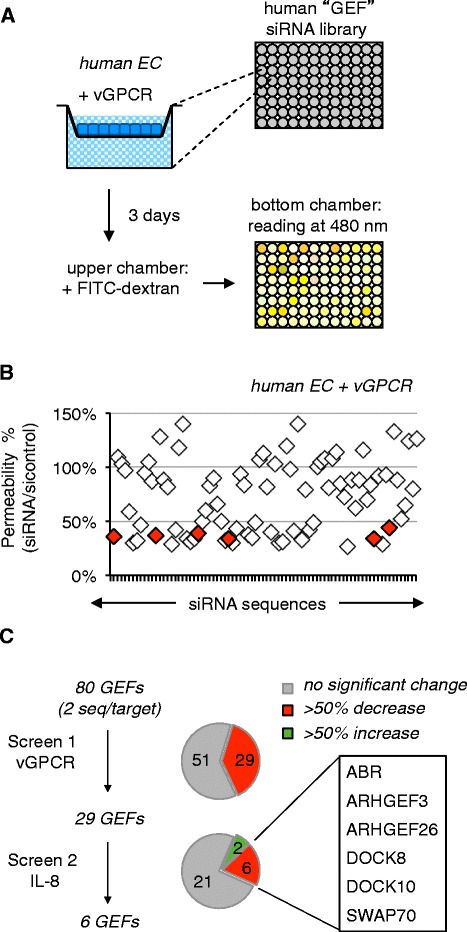
**A functional siRNA library screen identifies 6 putative GEF downstream of vGPCR. A**. Schematic of GEF siRNA permeability screen. **B**. The fluorescence intensity was determined for each targeting sequence (2 sequences/target) and expressed as means of the ratio to control siRNA-transfected cells (4 non-silencing control sequences). Shared hits between vGPCR and IL-8 screens are highlighted in red. **C**. A two-step screen was performed; the first examined vGPCR-driven permeability. Hits from that screen were then tested for involvement in IL-8-driven permeability. The six GEFs that significantly decreased permeability when silenced are indicated.

We aimed to identify molecular switches involved in Rac-dependent vGPCR-driven endothelial permeability. Because vGPCR hijacks IL-8-induced intracellular signaling pathways [1,4,10], we wanted to unmask GEFs that could contribute to both vGPCR and IL-8 actions. We thus screened a siRNA library against Rho family GEFs, which includes Rac GEFs, to pinpoint those required for the induction of permeability by vGPCR/IL-8. Using well-established methodologies, endothelial permeability was measured by the passage of FITC-conjugated 40 kDa dextran through a monolayer of human umbilical vein endothelial cells (HUVEC)-expressing vGPCR (Figure 1A; Additional file 1) [5,13]. The relative change in fluorescence intensity compared to control sequences (4 sequences/assay) was then determined for each GEF siRNA (2 sequences/target, 80 GEFs). Of the eighty GEFs screened, 29 were identified which, when silenced, decreased vGPCR-induced permeability by greater than 50% (Figure 1B; Additional file 2: Table S1). Hits from this first screen were subsequently tested in the context of IL-8-induced permeability (Figure 1C; Additional file 2: Table S1) [5,7]. Although two candidates increased permeability when silenced, the depletion of six others significantly reduced permeability directed by IL-8. The six GEFs that reduced both IL-8- and vGPCR-mediated permeability were ABR (active B cell receptor-related), ARHGEF3, ARHGEF26, dedicator of cytokinesis (DOCK) 8, DOCK10, and switch-associated protein 70 SWAP70 (Figure 1C). ARHGEF3, ARHGEF26, ABR, DOCK8 and DOCK10, which have not been reported for their ability to specifically activate Rac, were not considered at that stage. Of interest to us was the non-conventional Rac-GEF, SWAP70 [14]. This protein has been reported to be involved in various cellular processes including membrane ruffling, cell-cell adhesion, and migration, as well as cell proliferation and invasion [14-17]. SWAP70 itself is activated by the phosphoinositide 3K (PI3K) second messenger, phosphatidylinositol (3,4,5,) P_3_ (PIP3), which binds its pleckstrin homology (PH) domain and recruits the protein to the membrane [14]. Interestingly, both vGPCR and IL-8 were shown to elevate permeability through PI3K-dependent Rac activation [4,10], therefore placing SWAP70 as an appealing candidate.

**Figure 2:**
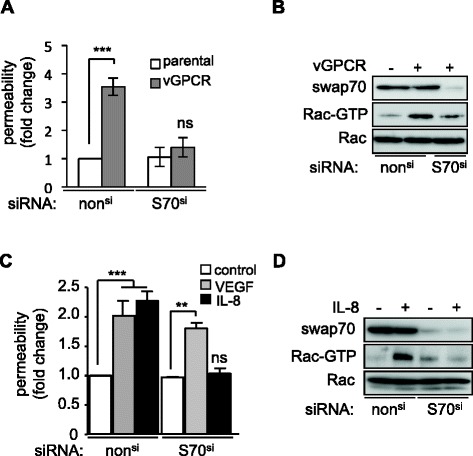
**SWAP70 modulates vGPCR- and IL-8-mediated permeability through Rac activation. A**. Permeability of SVEC (parental) or SVEC stably expressing vGPCR (vGPCR) that were transfected with non-silencing siRNA (non^si^) or siRNA against SWAP70 (S70^si^). **B**. Activated Rac was detected by Rac pulldown in cells with (+) and without (−) vGPCR expression following transfection with non^si^ or S70^si^. **C**. Permeability of non-stimulated SVEC (control) or those stimulated with either VEGF or IL-8 was determined after transfection S70^si^ or non^si^. **D**. Activated Rac was detected by Rac pulldown in cells with (+) and without (−) IL-8 stimulation following transfection with non^si^ or S70^si^. Data are representative of three independent experiments. ***p < 0.001; **p < 0.01; ns, non significant by analysis of variance (ANOVA).

We first confirmed that silencing SWAP70 impaired vGPCR-driven endothelial permeability using an independent siRNA sequence (Figure 2A). As expected, vGPCR expression conferred a significantly greater permeability index compared to that of the vGPCR-negative parental cell lines. In sharp contrast, this effect was lost when SWAP70 was silenced. We next investigated the impact of SWAP70 on Rac activation. Pull-down experiments revealed a strong activation of Rac in HUVEC-vGPCR cells, which was mitigated by silencing of SWAP70 (Figure 2B). Similarly, IL-8 failed to increase permeability and to mediate Rac activation in SWAP70-depleted cells in parental HUVEC (Figure 2C-D). In contrast, although vascular endothelial growth factor (VEGF)-driven permeability was impaired by Rac depletion [4] (Additional file 3: Figure S1), it was not affected by silencing of SWAP70 (Figure 2C). These data suggest that SWAP70 is necessary for vGPCR- and IL-8-driven endothelial permeability likely through its impact on Rac activation.

**Figure 3:**
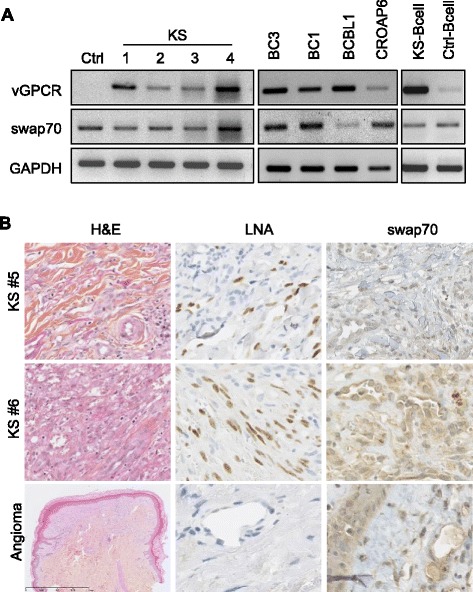
**SWAP70 is expressed in HHV8-infected cells and tissues. A**. Semi-quantitative PCR was used to analyze expression of vGPCR and SWAP70 in patient-derived non-KS (Ctrl) and KS skin lesions (KS #1 to #4), as well as in latently infected HHV8 cell lines (BC3, BC1, BCBL1, CROAP6), and patient-isolated KS-positive (KS-B cell) and –negative (Ctrl-B cell) peripheral B cells. GAPDH mRNA levels were detected as a control for input and equal loading. **B**. Immunohistochemical staining of hematoxylin/eosin (H&E) coloration, LNA, and SWAP70 in tissue sections from KS-positive lesions (KS #5 and KS #6) and a KS-negative lesion (angioma).

To examine whether SWAP70 expression may be associated with HHV8 infection, its gene expression levels were measured in KS patient-derived tissues, HHV8-positive cell lines, and KS patient-isolated peripheral B cells (Figure 3A). SWAP70 RNA was detected in four KS patient-derived skin lesions (Figure 3A, lanes 2–5) as well as in the non-KS skin lesion (Figure 3A, lane 1). Similarly, SWAP70 was present in four latently infected PEL cell lines (Figure 3A, lanes 6–9), as well as in both HHV8-positive and-negative patient peripheral B cells (Figure 3A, lanes 10–11). SWAP70 expression was also detected by immunohistochemistry in human-derived KS-positive lesions (as indicated by latent nuclear antigen (LNA) staining), as well as in non-KS, LNA-negative angioma (Figure 3B). These data demonstrate that SWAP70 is ubiquitously and homogeneously expressed regardless of the cell HHV8 status and highlight that SWAP70 is available for regulation of permeability mediated by vGPCR in HHV8-defined diseases.

**Figure 4:**
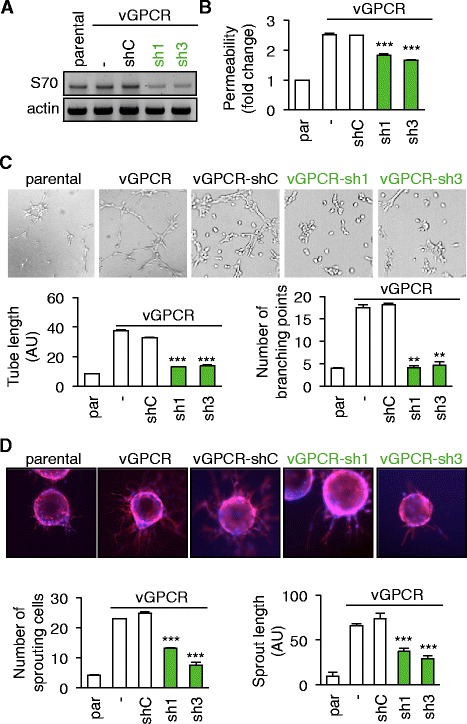
**SWAP70 is required to convey vGPCR pro-angiogenic actions in endothelial cells. A-D**. Parental (Par) and vGPCR-expressing SVEC (−) were stably transfected with control (shC) or two shRNAs against SWAP70 (sh1 and sh3) plasmids. **A**. Cells were processed for semi-quantitative RT-PCR to determine SWAP70 mRNA expression. Actin mRNA levels were detected as a control for input and equal loading. **B**. Permeability assays were performed as described in methods. **C-D**. SVEC cells were also tested for tube formation capacity (C) and sprouting ability (D). Representative images are shown. Tube length and number of branch points per field of view (FOV) were quantified in C. Number of sprouting cells and mean sprout length were determined in D. ***p < 0.001, **p < 0.01 by analysis of variance (ANOVA).

To further evaluate the role of SWAP70 in endothelial homeostasis in the context of vGPCR expression, previously established vGPCR-positive SV40-transformed mouse endothelial cells (SVEC-vGPCR) [4,11] were further engineered to stably express control- (shC) or SWAP70 targeting shRNAs. SWAP70 sh1 and sh3 sequences effectively impaired SWAP70 messenger expression, when compared with that of either the control (shC) or parental cell lines (Figure 4A) and were thus used in subsequent experiments. Further supporting our transient knockdown experiments in HUVEC, the stable silencing of SWAP70 in SVEC significantly impaired vGPCR-induced permeability when compared to shC endothelial lines (Figure 4B). As another hallmark of endothelial plasticity, the ability of SVEC to undergo *in vitro* tubulogenesis was tested under serum-free conditions [18]. While the parental SVEC cell line underwent limited tubulogenesis, the vGPCR-expressing cells readily invaded the matrigel and formed tubes (Figure 4C). Importantly, although stable transfection with the control shRNA had no effect on vGPCR-conferred tubulogenesis, this phenotype was visibly impaired in the SWAP70 knockdown-engineered sh1 and sh3 cell lines (Figure 4C). Quantitation of both tube length and the number of branching points further supported these findings (Figure 4C). Accordingly, the capacity of vGPCR-expressing SVEC to attach and sprout from matrigel-coated beads [19] was also significantly negatively impacted by SWAP70 silencing (Figure 4D). As a comparison, parental SVEC sprouting abilities were clearly limited. Notably, numbers of sprouting cells, as well as the mean sprout length, were diminished in SWAP70-silenced cells (sh1 and sh3), but not in those of the control shRNA (shC), when compared to SVEC-vGPCR (Figure 4D). These data therefore suggest that SWAP70 is necessary for the vGPCR-promoted angiogenic phenotype of endothelial cells.

In this study, SWAP70 was identified as a protein required for vGPCR-driven endothelial permeability through a genetic screen using siRNAs against 80 referenced human GEFs. In keeping with its conserved role as a GEF [14,20], it was indeed found that silencing SWAP70 impaired Rac activation mediated by vGPCR, suggesting that SWAP70 functions downstream of vGPCR to modulate the Rac activation state. SWAP70 was also necessary for IL-8-driven endothelial permeability and Rac activation. Because IL-8 signals through CXCR2, the human homologue of vGPCR, this suggests that vGPCR fully mimicked CXCR2 signaling by pirating the SWAP70/Rac axis in endothelial cells [4,10]. As mentioned earlier, SWAP70 recruitment to the plasma membrane, which is a prerequisite for GEF enzymatic activity, involves the PI3K second messenger PIP3 [14]. Both vGPCR and CXCR2 drive endothelial permeability via the PI3K-Rac signaling nexus [4,10,11], SWAP70 might therefore fit downstream of PI3K in vGPCR/CXCR2-driven permeability pathway activation.

SWAP70 was also necessary for vGPCR-mediated endothelial tubulogenesis and sprouting. These two assays measure the migratory and invasive capacity of endothelial cells as an indicator of angiogenic potential and plasticity. Thus, SWAP70 appears to be essential for the angiogenic phenotype imposed on endothelial cells by vGPCR [1,2,7]. In keeping with this idea, SWAP70 also regulates migration and invasion in various immune cells [15,21,22]. In dendritic cells, for example, it transmits migratory and invasive signals elicited by sphingosine-1 phosphate [23]. SWAP70 also influences these processes in glioma and prostate cancer cells [24,25]. In these cases, depletion of SWAP70 correlated with both reduced cell migration and invasion [24,25]. Conversely, co-expression of SWAP70 with oncogenic v-Src in mouse embryonic fibroblasts has been shown to result in a more aggressive and invasive phenotype than when v-Src is expressed alone [17]. Thus although SWAP70 is widely expressed and functional, it seems that its deregulation as a result of its exploitation by oncogenes, in this case vGPCR, could promote the development of more aggressive tumors.

It should be mentioned that five additional GEFs were identified in the screen that also impaired vGPCR/IL-8-induced permeability. Owing to their lack of reported Rac GEF activity, they were not further explored. However, it is interesting to speculate that they potentially play additive roles in vGPCR-promoted permeability via Rac-independent pathways. Thus their role in vGPCR-promoted permeability should be deciphered in future studies.

In summary, this study identifies the Rac GEF SWAP70 as a mediator of permeability downstream of both vGPCR and IL-8. Blockade of SWAP70 function could be of therapeutic value to prevent permeability and activation of endothelial cells and thus hinder tumor angiogenesis.

## Availability of supporting data

The data sets and methods description supporting the results of this article are included within the article and its Additional file 1, Additional file 2: Table S1 and Additional file 3: Figure S1.

## Additional files

Additional file 1:
**Materials and Methods.**
Additional file 2: Table S1. GEF siRNA library screen on vGPCR- and IL-8-dependent endothelial permeability increase. vGPCR-expressing and IL-8 stimulated (50 ng/ml, 1h) human umbilical vein endothelial cells (HUVEC) were transfected with 4 different non silencing RNA (nonsi) as controls or 2 different sequences targeting GEF (GEFsi, Qiagen, 50 nM). Three days later, permeability was assessed as described in methods. Results were expressed as the percentage of the values obtained for GEFsi (*2) normalized to the mean of values obtained for nonsi conditions (*4). Shared hits are highlighted in green. Additional file 3: Figure S1. Effect of Rac silencing on VEGF-induced permeability. **A.** Permeability of SVEC that were transfected with non-silencing siRNA (nonsi) r siRNA against Rac1 (Racsi). **B.** Silencing of Rac1 was confirmed by western-blot in total lysates, 3 days post-transfecEon.

## Abbreviations

ABR: Active B cell receptor-related
DOCK: Dedicator of cytokinesis
GEF: Guanine exchange factor
GTP: Guanosine triphosphate
HHV8: Human herpes virus 8
HUVEC: Human umbilical vein endothelial cell
KS: Kaposi sarcoma
KSHV: Kaposi sarcoma herpes virus
LNA: Latent nuclear antigen
PEL: Primary effusion lymphoma
PH: Pleckstrin homology
PI3K: Phosphoinositide 3 kinase
shRNA: Small hairpin RNA
SWAP70: Switch-associated protein 70
VEGF: Vascular endothelial growth factor
vGPCR: Viral G-protein coupled receptor
