# Supplementary material for: The guanine exchange factor SWAP70 mediates vGPCR-induced endothelial plasticity
Source: Cell Commun Signal. 2015 Feb 15;13:11. doi: 10.1186/s12964-015-0090-1 (PMC4336709; doi:10.1186/s12964-015-0090-1)
Supplement: Additional file 1: — Materials and Methods. [file 12964_2015_90_MOESM1_ESM.docx]

**Materials and Methods**

**Cell culture**

HEK-293T, SVEC4-10 and HUVEC Eahy.926 cell lines were obtained from the ATCC and maintained in DMEM supplemented with 10% fetal bovine serum (FBS) and 1% penicillin/streptomycin (all from Life Technologies). BC3, BC1, BCBL1 and CROAP6 PEL lines were purchased from DMSZ and cultured in RPMI supplemented with 20% FBS and 1% penicillin/streptomycin (all from Life Technologies). G418-selected stable SVEC4-10 and HUVEC cell lines were obtained by transfection of pCEFL-vGPCR as described previously [1].

**Human tissues and samples**

After giving their informed consent, skin and peripheral blood specimens were collected from patients with KS or non-KS skin lesions (Sexually Transmitted Disease Center, Pavillon Tarnier, Paris, France). Skin biopsies were split into two parts; one was snap-frozen and conserved at -80°C until RT-PCR analysis and the other was formalin-fixed and paraffin-embedded for immunohistochemical analysis. Peripheral B-cells were isolated as described in [2].

**Immunohistochemistry**

For immunohistochemistry, thick sections (4 µm) were cut and stained with hematoxylin and eosin (H&E). After heat-induced epitope retrieval, the avidin–biotin peroxidase complex technique (kit A. Menarini Diagnostics), was used to detect LNA and SWAP70. Primary polyclonal antibodies were diluted at 1:100 in S2022 diluent (Dako). Results were blindly analyzed using an optical microscope (original magnifications x40).

**siRNA transfection and GEF siRNA library**

siRNA library was customly designed from Qiagen to target 80 human GEFs (Additional file 2). Library contains 2 sequences/target, as well as four negative controls. For transient SWAP70 gene silencing in HUVEC, siRNA against SWAP70 (CAGGAGAGCUUUACGCUAA) was purchased from Dharmacon (Thermofisher Scientific). Non-silencing control siRNA was from Life Technologies.

**shRNA retroviral transduction**

For stable knockdown of SWAP70 in SVEC, lentiviral particles were produced as in [3]. Briefly, HEK-293T were transfected by calcium phosphate with a mixture of pVSV, pSPAX2 and pGIPZ-GFP or pGIPZ-GFP-SWAP70 shRNA (Thermofisher Scientific). Two days post-transfection, supernatants were collected and prepared as described in [3].

**Reagents and Antibodies**

Recombinant human IL-8 and VEGF were purchased from PeproTech. SWAP70 antibodies were from Abnova, Rac1 antibodies from BD Biosciences, and LNA from Novacostra. Secondary antibodies were from Life Technologies.

**GST Pull-downs and Western-Blots**

Cells were transfected with non-silencing or SWAP70-targetting sequences, serum-starved three days later and protein lysates were extracted in 10 mM Tris-HCl pH 7.5, 110 mM NaCl, 1 mM EDTA, 10 mM MgCl2, 1% Triton X-100, 10 mM β-glycerophosphate. Post-nuclei supernatants were saved for protein concentration measurement (microBCA, Pierce) and western blotting for the total expression of Rac and SWAP70. Equal amount of proteins were further processed for GST pull-down experiments as described in [1].

**RT-PCR**

RT-PCR was conducted as described in [4]. The mRNA expression of the following genes was examined using the indicated primer sets: human SWAP70 (tttctgcccagccatttatc, aaggccacactgtttccatc), mouse SWAP70 (AGCCATGTACGTAGCCATCC, CTCTCAGCTGTGGTGGTGAA), human actin (agcactgtgttggcgtacag, ggacttcgagcaagagatgg), mouse actin (TCCACCATCCATCTGTTGAA, GCGTTTCTTTTCCTCGTCTG), human GAPDH (GAGTCAACGGATTTGGTCGT, TTGATTTTGGAGGGATCTCG) and vGPCR (TGTGTGGTGAGGAGGACAAA, GTTACTGCCAGACCCACGTT).

**Permeability, Tubulogenesis and Sprouting Assays**

*In vitro* permeability, tubulogenesis and sprouting assays were conducted as described previously in [5], [4], and [6], respectively.

**References**

1. Dwyer J, Le Guelte A, Galan Moya EM, Sumbal M, Carlotti A, Douguet L, Gutkind JS, Grange PA, Dupin N, Gavard J: **Remodeling of VE-cadherin junctions by the human herpes virus 8 G-protein coupled receptor.** *Oncogene* 2011, **30:**190-200.

2. Azzi S, Smith SS, Dwyer J, Leclair HM, Alexia C, Hebda JK, Dupin N, Bidere N, Gavard J: **YGLF motif in the Kaposi sarcoma herpes virus G-protein-coupled receptor adjusts NF-kappaB activation and paracrine actions.** *Oncogene* 2014, **33**:5609-18.

3. Dubois SM, Alexia C, Wu Y, Leclair HM, Leveau C, Schol E, Fest T, Tarte K, Chen ZJ, Gavard J, Bidere N: **A catalytic-independent role for the LUBAC in NF-kappaB activation upon antigen receptor engagement and in lymphoma cells.** *Blood* 2014, **123:**2199-2203.

4. Dwyer J, Hebda JK, Le Guelte A, Galan-Moya EM, Smith SS, Azzi S, Bidere N, Gavard J: **Glioblastoma cell-secreted interleukin-8 induces brain endothelial cell permeability via CXCR2.** *PLoS One* 2012, **7:**e45562.

5. Gavard J, Gutkind JS: **VEGF controls endothelial-cell permeability by promoting the beta-arrestin-dependent endocytosis of VE-cadherin.** *Nat Cell Biol* 2006, **8:**1223-1234.

6. Bignon M, Pichol-Thievend C, Hardouin J, Malbouyres M, Brechot N, Nasciutti L, Barret A, Teillon J, Guillon E, Etienne E, et al: **Lysyl oxidase-like protein-2 regulates sprouting angiogenesis and type IV collagen assembly in the endothelial basement membrane.** *Blood* 2011, **118:**3979-3989.
