# Supplementary material for: The guanine exchange factor SWAP70 mediates vGPCR-induced endothelial plasticity
Source: Cell Commun Signal. 2015 Feb 15;13:11. doi: 10.1186/s12964-015-0090-1 (PMC4336709; doi:10.1186/s12964-015-0090-1)
Supplement: Additional file 2: Table S1. — GEF siRNA library screen on vGPCR- and IL-8-dependent endothelial permeability increase. vGPCR-expressing and IL-8 stimulated (50 ng/ml, 1h) human umbilical vein endothelial cells (HUVEC) were transfected with 4 different non silencing RNA (nonsi) as controls or 2 different sequences targeting GEF (GEFsi, Qiagen, 50 nM). Three days later, permeability was assessed as described in methods. Results were expressed as the percentage of the values obtained for GEFsi (*2) normalized to the mean of values obtained for nonsi conditions (*4). Shared hits are highlighted in green. [file 12964_2015_90_MOESM2_ESM.pdf]

Gene	Permeability fold (GEFsi/nonsi) in vGPCR cells	Permeability fold (GEFsi/nonsi) in IL8-stimulated cells
ABR	36%	33%
AKAP13	109%	107%
ALS2	103%	60%
ARHGEF10	97%	127%
ARHGEF10L	59%	79%
ARHGEF15	30%	95%
ARHGEF16	32%	145%
ARHGEF17	47%	95%
ARHGEF18	95%	90%
ARHGEF19	105%	93%
ARHGEF2	87%	90%
ARHGEF3	37%	47%
ARHGEF4	128%	111%
ARHGEF5	88%	96%
ARHGEF9	82%	87%
BCR	29%	53%
C9orf100	42%	78%
COOL1	118%	120%
COOL2	140%	134%
Dbl	34%	122%
Dbs	31%	98%
DOCK1	34%	58%
DOCK10	39%	40%
DOCK11	50%	60%
DOCK2	60%	82%
DOCK3	83%	64%
DOCK4	90%	87%
DOCK5	66%	72%
DOCK6	50%	102%
DOCK7	32%	73%
DOCK8	34%	47%
DOCK9	30%	62%
ECT2	43%	81%
FARP1	94%	118%
FARP2	84%	94%
FGD1	38%	106%
FGD2	35%	82%
FGD3	49%	104%
FGD5	107%	139%
FGD6	113%	98%
FLJ43963	82%	94%
Frabin	40%	174%
GEFT	103%	117%
ITSN1	30%	75%
ITSN2	31%	169%
KALRN	120%	127%
KALRN	98%	104%

KIAA1909	41%	89%
LARG	140%	112%
LOC345930	33%	54%
MCF2L2	79%	96%
NET1	42%	50%
NGEF	49%	92%
OBSCN	100%	115%
p115	105%	95%
PDZ-GEF	108%	124%
PLEKHG1	81%	105%
PLEKHG2	108%	112%
PLEKHG3	114%	136%
PLEKHG4	85%	95%
PLEKHG5	73%	49%
PLEKHG6	27%	84%
PREX1	88%	120%
PREX2	62%	79%
RASGRF1	88%	87%
RASGRF2	116%	102%
RGNEF	70%	104%
Scambio	84%	79%
SGEF	34%	33%
SOS1	92%	82%
SOS2	29%	75%
SPATA13	93%	128%
SWAP70	44%	41%
TIAM1	133%	133%
TIAM2	88%	134%
TRIO	52%	88%
Tuba	65%	97%
VAV1	124%	111%
VAV2	80%	126%
VAV3	126%	125%

Table 1. GEF siRNA library screen on vGPCR- and IL-8-dependent endothelial permeability increase.
vGPCR-expressing or IL-8 stimulated (50 ng/ml, 1h) human umbilical vein endothelial cells (HUVEC) were transfected with 4 different non silencing RNA sequences (nonsi) as controls, or 2 different sequences targeting the 80 GEFs (GEFsi, Qiagen, 50 nM). Three days later, permeability was assessed as described in methods. Results were expressed as the percentage of the values obtained for GEFsi (*2) normalized to the mean of values obtained for nonsi conditions (*4). Positive hits (decrease greater than 50%) are highlighted in green.
