# Supplementary material for: The guanine exchange factor SWAP70 mediates vGPCR-induced endothelial plasticity
Source: Cell Commun Signal. 2015 Feb 15;13:11. doi: 10.1186/s12964-015-0090-1 (PMC4336709; doi:10.1186/s12964-015-0090-1)
Supplement: Additional file 3: Figure S1. — Effect of Rac silencing on VEGF-induced permeability. A. Permeability of SVEC that were transfected with non-silencing siRNA (nonsi) r siRNA against Rac1 (Racsi). B. Silencing of Rac1 was confirmed by western-blot in total lysates, 3 days post-transfecEon. [file 12964_2015_90_MOESM3_ESM.pdf]

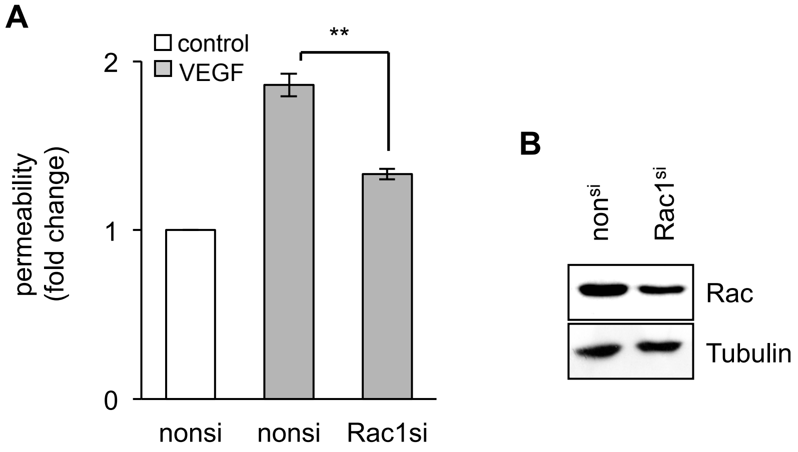

Figure S1. Effect of Rac silencing on VEGF-induced permeability.

A. Permeability of SVEC that were transfected with non-silencing siRNA (non^{si}) or siRNA against Rac1 (Rac^{si}). **B.** Silencing of Rac1 was confirmed by western-blot in total lysates, 3 days post-transfection.
